# Eliminating Meningococcal Epidemics From the African Meningitis Belt: The Case for Advanced Prevention and Control Using Next-Generation Meningococcal Conjugate Vaccines

**DOI:** 10.1093/infdis/jiz297

**Published:** 2019-10-31

**Authors:** Mark R Alderson, F Marc LaForce, Ajoke Sobanjo-ter Meulen, Angela Hwang, Marie-Pierre Preziosi, Keith P Klugman

**Affiliations:** 1 Center for Vaccine Innovation and Access, PATH; 2 Bill & Melinda Gates Foundation, Seattle, Washington; 3 Angela Hwang Consulting, Albany, California; 4 Technical Services, Serum Institute of India Pvt Ltd, Pune, India; 5 Immunization, Vaccines, and Biologicals, World Health Organization, Geneva, Switzerland

**Keywords:** meningitis belt, multivalent meningococcal vaccines, epidemic prevention

## Abstract

The introduction and rollout of a meningococcal serogroup A conjugate vaccine, MenAfriVac, in the African meningitis belt has eliminated serogroup A meningococcal infections for >300 million Africans. However, serogroup C, W, and X meningococci continue to circulate and have been responsible for focal epidemics in meningitis belt countries. Affordable multivalent meningococcal conjugate vaccines are being developed to prevent these non-A epidemics. This article describes the current epidemiologic situation and status of vaccine development and highlights questions to be addressed to most efficiently use these new vaccines.

Epidemic meningococcal disease has long been an important public health problem in sub-Saharan Africa. Until 2011, the majority of meningococcal disease epidemics in the African meningitis belt were caused by serogroup A *Neisseria meningitidis* [1, 2]. To address this problem, an affordable monovalent meningococcal serogroup A conjugate vaccine (MACV), MenAfriVac, was developed specifically for African populations. Given the magnitude of serogroup A meningitis epidemics and the high rates of endemic serogroup A disease in meningitis belt countries, the World Health Organization (WHO) recommended an MACV deployment strategy, starting with mass vaccination campaigns for 1–29-year-olds to rapidly establish herd protection and control serogroup A disease, followed by routine immunization of infants and toddlers to sustain this protection and prevent a resurgence of epidemics [3].

After licensure and WHO prequalification of MenAfriVac, vaccine campaigns began in December 2010 in Burkina Faso, Mali, and Niger. The vaccine was well received, with coverage rates >90%, and by the middle of 2011 it was clear that the vaccine was having a major impact on serogroup A carriage and disease. Post introduction meningitis surveillance revealed that serogroup A meningococcal disease had disappeared in all age groups, not just those that received the vaccine, strongly suggesting that robust herd immunity had been achieved [4]. Over the next 8 years, >300 million Africans were immunized, and serogroup A meningococcal infections virtually disappeared wherever the vaccine was given. Starting in 2016, meningitis belt countries began introducing MACV the serogroup A conjugate vaccine into their routine immunization programs [5, 6].

## CONTINUED PROBLEMS WITH NON-A MENINGOCOCCAL EPIDEMICS

Epidemics due to serogroups C, W, and X meningococci have continued in meningitis belt countries after MACV introduction. The most serious epidemics have been due to serogroup C *N. meningitidis* and have occurred in Nigeria and Niger from 2014 to 2017. In the meningitis belt, the historical response to meningococcal disease epidemics has been to conduct reactive vaccination campaigns once an outbreak is identified. Since 1997, the International Coordination Group on Vaccine Provision for Epidemic Meningitis (ICG) has managed security stocks of vaccines for global emergency use and distributed meningococcal vaccines to African countries in response to meningitis epidemics. More than 4 million doses of serogroup C–containing meningococcal vaccines were distributed in Niger and Nigeria to combat these outbreaks [7]. 

In addition, serogroup W has been implicated in large meningococcal epidemics in Africa and serogroup X has emerged with the potential to cause meningitis epidemics, with increasing cases being reported in Burkina Faso, Chad, Mali, Niger, Nigeria, and Togo [8, 9]. Serogroup Y, although frequently found in carriage surveys, has not yet been a significant cause of disease. No carriage or cases due to meningococcus B have been reported in meningitis belt countries. However, serogroup B could become a problem in the future, and if so an alternative strategy using protein vaccines will be required [10, 11].

Reactive vaccination campaigns often begin in the late stages of an epidemic and can only prevent a minority of cases. Moreover, the ICG is facing increasingly serious vaccine supply challenges. Until recently, it has relied primarily on a few vaccine manufacturers able to supply affordable meningococcal polysaccharide vaccines. Most manufacturers have now shifted to producing meningococcal conjugate vaccines (NmCVs), which are superior owing to their ability to induce immunologic memory, generate herd immunity, and effectively immunize children <2 years old [12]. Three 4-valent NmCVs that target serogroups A, C, W, and Y have been licensed and prequalified by the WHO (Menactra, Menveo, and Nimenrix); however these vaccines are significantly more expensive than polysaccharide vaccines [13, 14]. With this shift to conjugate vaccines, the supply of meningococcal polysaccharide vaccines has diminished, and the ICG has had well-publicized difficulties in obtaining affordable vaccines to address non-A epidemics [15, 16].

A multivalent NmCV that is affordable could be used to prevent non-A epidemics in the meningitis belt, following the MACV example. In addition, sufficient supplies of a multivalent NmCV would facilitate epidemic response and could be the basis for a revolving stockpile with greater efficiency and less waste than the current meningococcal vaccine stockpile. New, potentially more affordable 4–5-valent NmCVs that are being developed have the potential to fill these gaps.

## NEW MULTIVALENT VACCINES FOR THE MENINGITIS BELT

Because meningococcal epidemics are unpredictable, impose a serious long-term burden on affected households, can severely disrupt health systems, and generate fear and confusion in affected countries, ministries of health and expert policy advisors aspire to prevent non-A epidemics, just as the serogroup A epidemics have been prevented [17–19]. In response, the partnership between PATH and the Serum Institute of India Pvt Ltd (SIIPL) that enabled the development, licensure, and introduction of MenAfriVac is now developing an affordable 5-valent NmCV (NmCV-5) for serogroups A, C, W, X and Y with financial support provided by the UK Department for International Development.

Initial development of the SIIPL NmCV-5 reduced manufacturing costs by improving the efficiency of carrier protein production, polysaccharide fermentation and purification, and chemical conjugation. One important component of this NmCV-5 is serogroup X, against which there are no licensed vaccines. The PATH-SIIPL collaboration has developed a high-yield serogroup X–tetanus toxoid conjugate that is highly immunogenic in preclinical animal models [20]. For the 5-valent vaccine formulation, serogroup A and X polysaccharides are conjugated to tetanus toxoid, and serogroup C, W, and Y polysaccharides are conjugated to recombinant CRM_197_, a genetically altered form of diphtheria toxin that lacks toxin activity.

A recently completed phase 1 trial of SIIPL’s NmCV-5 in healthy US adults (18 – 45 years of age) demonstrated that the vaccine is safe and well tolerated and elicits functional immune responses (rabbit complement serum bactericidal activity) predicted to confer protection against all 5 targeted serogroups [21]. A phase 2 study in toddlers (aged 12–16 months) in Mali was designed to confirm safety and immunogenicity and to select a formulation (adjuvanted or nonadjuvanted) for future trials. That study confirmed that SIIPL’s NmCV-5 is safe and immunogenic against all serogroups (preliminary results). In addition, use of an aluminum phosphate adjuvant did not improve immunogenicity; hence, phase 3 formulations of NmCV-5 will be nonadjuvanted. Phase 3 trials in Africa and India are anticipated to begin in mid-2019. Licensure will be based on demonstration of adequate safety and immunologic noninferiority to a licensed and WHO-prequalified 4-valent NmCV for serogroups A, C, W, and Y. The regulatory strategy for the SIIPL NmCV-5 involves initial licensure in India based on the planned phase 3 trials and then submission to WHO for prequalification to facilitate availability to countries in the African meningitis belt.

In contrast to MACV, which was designed specifically for the meningitis belt, there is a global market for multivalent NmCVs, and, as a result, multiple developing country vaccine manufacturers are pursuing lower cost NmCVs. Provided they conform to international quality standards and meet WHO prequalification criteria, these vaccines could provide valuable supply security for meningitis belt countries and make meningitis prevention more widely available in other regions as well. These vaccines are generally in early-stage clinical development in countries such as China, India, and Brazil and could potentially be available to global markets, including the African meningitis belt, within a few years of NmCV-5.

## STRATEGIES TO DEPLOY NEW MULTIVALENT MENINGOCOCCAL CONJUGATE VACCINES IN AFRICA

Replicating the broad introduction of MACV across the 26 countries of the meningitis belt with multivalent NmCVs is likely to present financial challenges owing to the higher cost of manufacturing multivalent vaccines, budgetary constraints, and competing priorities for ministries of health and funding agencies, such as Gavi, the Vaccine Alliance. In November 2018, Gavi approved in principle the expansion of its existing meningococcal program to support serogroup ACW-containing NmCVs, provided that policy and regulatory requirements are satisfied and cost and impact assumptions are met [22]. Targeted, efficient vaccine deployment strategies tailored for different areas of the meningitis belt will be needed to make the most effective use of limited financial resources.

There are 2 broad groups of countries in the African meningitis belt differentiated by meningococcal disease endemicity, which could adopt different NmCV vaccination strategies. The first includes high- and medium-incidence countries, such as Burkina Faso, Cameroon, Chad, The Gambia, Mali, Niger, and Sudan, and subnational regions, such as northern areas of Nigeria, Ghana, and Togo and western Ethiopia, that have historically had high baseline rates of meningococcal infections and experienced large, unpredictable meningitis epidemics. The second includes a group of countries with historically lower rates of meningococcal disease that are adjacent to hyperendemic countries and have areas of high epidemic risk and occasional meningitis epidemics on their shared borders. Benin, Burundi, the Central African Republic, Côte d’Ivoire, the Democratic Republic of the Congo, Eritrea, Guinea, Guinea-Bissau, Kenya, Mauritania, Rwanda, Senegal, South Sudan, Tanzania, and Uganda fall into this second category [6, 13].

In high- and medium-incidence countries, the implementation strategy for multivalent NmCVs could emulate that of MACV for rapidly achieving and efficiently maintaining herd immunity. Carriage was found to be highest during childhood and adolescence (among 5–14-year-olds), and strategies combining routine immunization and single-dose campaigns targeting the high transmitters have a potentially high impact [23]. Single doses of multivalent NmCV delivered to the age groups that contribute most to transmission could rapidly abolish carriage of serogroups C, W, X and Y, in addition to serogroup A, thereby generating herd protection. Routine immunization or periodic mass campaigns with multivalent NmCVs would then be needed to sustain population protection. Such strategies are of interest to hyperendemic countries because they offer the promise of ending all meningococcal meningitis outbreaks.

Lower-risk countries may choose alternative strategies. At the 2017 African Meningitis Meeting in Ouagadougou, Burkina Faso, representatives from lower-risk countries were asked informally how they might use a serogroup ACWXY NmCV. Some representatives expressed a preference for substituting a multivalent NmCV for the monovalent MACV currently used in routine immunization programs. Others wished to continue with MACV, provided that a stockpile of multivalent NmCVs is available for rapid use should a meningitis epidemic occur.

Emergency access to multivalent NmCVs will still be needed for countries in the meningitis belt that do not routinely vaccinate with multivalent NmCVs and in the event of *N. meningitidis* outbreaks outside currently identified high-risk areas. Unlike with polysaccharide vaccines, unused doses of stockpiled NmCVs could be repurposed for preventive use on a global, regional, or national basis. In the mid-term future, sufficient affordable multivalent NmCVs should ideally be available globally to respond to outbreaks wherever they occur, so that meningitis vaccine stockpiles are no longer needed.

## STUDIES TO INFORM THE USE OF MULTIVALENT MENINGOCOCCAL CONJUGATE VACCINES

Studies described elsewhere in this supplement have yielded a more detailed understanding of meningococcal epidemiology, transmission and immunity, creating an opportunity to consider targeted strategies with the potential to achieve similar outcomes to MACV but with greater efficiency. For example, mass vaccination campaigns could focus on a more limited age group, such as 2–19-year-olds, rather than 1–29-year-olds, to prevent epidemics through herd protection. Routine immunization could target the age groups that give the best combination of coverage and duration of immunity. To design efficient multivalent NmCV immunization strategies, several questions must be addressed:

- How does multivalent NmCV immunization affect age-specific carriage prevalence?
- How does multivalent NmCV immunization in a given age group affect transmission across the entire population?
- Would mass campaigns targeting a narrower age range, such as 2–19 or 2–14 years, be acceptable to communities? What alternative age ranges might be preferred?
- What would be the optimal routine immunization schedule for multivalent NmCVs, in terms of target ages and numbers of doses? Will immunizing infants and toddlers give sufficient duration of protection to maintain herd immunity? If not, what are the best alternatives or complements to infant/toddler vaccination?

Two studies are envisaged to answer many of these questions. First, a pivotal phase 3 trial of SIIPL’s NmCV-5 in 9–18-month-old children would evaluate immune responses and antibody persistence in vaccinated subjects. Second, a study in at least 2 countries (Burkina Faso and Niger) could evaluate how immunizing a range of different age groups with NmCV-5 affects carriage in nonvaccinated community members. This would result in direct comparison of the different strategies and their ability to generate herd protection.

To inform vaccine introduction strategies and policy decisions, results from these studies and complementary surveillance and other data need to be incorporated into models that explore the tradeoffs between age at vaccination, vaccination coverage, antibody persistence, herd immunity, and cost effectiveness. These models can simulate a wide range of options and compare the health and economic effects of different immunization strategies as a basis for WHO policy recommendations and Gavi decision making.

These studies would build on substantial evidence from research that documented the effect of vaccination on carriage of serogroup A and C meningococci. Carriage studies done during the introduction of MACV indicated that serogroup A meningococcal carriage was virtually eliminated in vaccinated communities, with a 98% decrease in carriage prevalence observed in Chad among nonvaccinated persons [24–26]. This led to herd protection such that all persons, vaccinated and nonvaccinated, were protected from serogroup A infection. Surveys conducted during and following introduction of serogroup C NmCV routine immunization in persons aged 15–19 years in the United Kingdom showed significant decreases in carriage of serogroup C concurrent with dramatic decreases in serogroup C meningitis in nonvaccinated as well as vaccinated individuals [27].

Communities grappling with meningitis have insistently called for a global action plan for “defeating meningitis by 2030,” in line with the commitment of the United Nations Sustainable Development Goals, “To transform our world for the better by 2030” [28, 29]. If studies with NmCV-5 show that it significantly decreases serogroup C, W, X, and Y carriage, it will be strong evidence that the new multivalent vaccine has the potential to transform meningitis control in Africa by dramatically reducing the incidence of meningococcal disease and eliminating all major causes of meningococcal outbreaks in vaccinated communities.

## Notes

Presented in part: “Assessing the Impact of a Multivalent Meningococcal Conjugate Vaccine on Meningococcal Carriage in Africa,” Norwegian Institute of Public Health, Oslo, April 23–24, 2018; “Toward a Multivalent Meningococcal Conjugate Vaccine,” Bill & Melinda Gates Foundation, Seattle, Washington, November 5, 2018.

## Notes

***Acknowledgments.*** We thank the organizers and participants of those meetings for sharing their time and insights. Special thanks go to Dominique Caugant and Paul Kristiansen, Norwegian Institute of Public Health; Caroline Trotter, University of Cambridge; Ryan Novak and LeAnne Fox, US Centers for Disease Control and Prevention; Matthew Coldiron, Epicentre (MSF); James Stuart, University of Bristol; André Bita, World Health Organization; Niranjan Bhat, PATH; Peter Dull, Bill & Melinda Gates Foundation; Ray Borrow, Public Health England; and Brian Greenwood, London School of Hygiene and Tropical Medicine.

***Financial support.*** This work was funded by the MenAfriNet consortium (www.menafrinet.org) through a grant from the Bill & Melinda Gates Foundation (OPP1084298).

***Potential conflicts of interest.*** M. R. A. reports grants from the UK Department of International Development during the conduct of the study. F. M. L. reports other support from PATH, Seattle, Washington, and grants from the UK Department of International Development during the conduct of the study, as well as personal fees from the Serum Institute of India Pvt Ltd outside the submitted work. A. H. reports personal fees from the Bill & Melinda Gates Foundation during the conduct of the study. M. P. P. reports grants from the Bill & Melinda Gates Foundation and Gavi, the Vaccine Alliance during the conduct of the study. All other authors report no potential conflicts. All authors have submitted the ICMJE Form for Disclosure of Potential Conflicts of Interest. Conflicts that the editors consider relevant to the content of the manuscript have been disclosed.
